# Allergen sensitization study in Dongying, China: An epidemiological study

**DOI:** 10.1097/MD.0000000000036862

**Published:** 2024-01-19

**Authors:** Yingying Zhang, Mei Shang, Ying Tian, Xuwei Liu, Xuhui Sun, Lianjun Gao

**Affiliations:** a Department of Pediatrics, Shengli Oilfield Central Hospital, Dongying City, Shandong Province, China.

**Keywords:** allergen sensitization, food allergens, pollen, specific IgE (sIgE)

## Abstract

**Background:**

To explore the relationship between specific immunoglobulin E levels in response to prevalent pollen and food allergens among patients suffering from localized allergic diseases in the Dongying area of China, and to analyze the interconnectivity among these factors.

**Methods:**

This research encompassed allergic patients who visited the Allergy Department of Shengli Oilfield Central Hospital from January 2022 to January 2023. We examined the specific immunoglobulin E levels in the blood of 230 patients utilizing the Fobock platform provided by Jiangsu Haoeubo Company. Statistical analysis was conducted with SPSS 25.0 statistical software. The chi-square test evaluated the relevance of differences in gender and age. A value of *P* < .05 was considered statistically significant.

**Results:**

In this study, eggs emerged as the allergen with the highest number of sensitized individuals, closely followed by dust mite. Conversely, the least sensitized allergen was the cypress tree, closely followed by mango. Notably, male patients exhibited higher sensitivities to cottonwood (*P* < .05) and egg (*P* < .001) compared to female patients. Children aged 0 to 10 years showed increased sensitivity to variety of allergens. A significant correlation was observed among different allergens. The top ten allergen pairs with the highest correlation included Birch Tree and Cottonwood (0.88, *P* < .001), Cottonwood and Pine Tree (0.86, *P* < .001), Birch Tree and Pine Tree (0.84, *P* < .001), Pine Tree and Paulownia (0.81, *P* < .001), Dust Mite and House Dust Mite (0.76, *P* < .001), Birch Tree and Paulownia (0.73, *P* < .001), Cashew and Pistachio (0.71, *P* < .001), Apple and Hazelnut (0.71, *P* < .001), Cottonwood and Paulownia (0.71, *P* < .001), and Pine Tree and Ordinary Ragweed (0.70, *P* < .001).

**Conclusion:**

This research sheds light on the patterns of allergen sensitization in Dongying, Shandong, highlighting that egg is the most prevalent sensitizing allergen. A notably high correlation was observed between Birch Tree and Cottonwood. This study enhanced the understanding of allergic diseases, explored the causes and mechanisms of allergies, strengthened the management of allergic diseases. Furthermore, it offers valuable insights for the clinical diagnosis and prevention of allergic diseases.

## 1. Introduction

Hypersensitivity conditions, a group of common disorders driven by the immune system, include a range of ailments such as hay fever (Allergic Rhinitis, AR), bronchial asthma, ocular allergies, eczema (Atopic Dermatitis, AD), and reactions to certain foods. These conditions typically originate from the body’s immunoglobulin E (IgE) responses to harmless external substances, or allergens, with the specific response depending on the allergen’s point of contact with the body.^[1]^ Diagnostic techniques involve detecting specific IgE (sIgE) antibodies to these allergens either through skin (dermal puncture tests) or by identifying sIgE in blood samples (blood-sIgE assessments).^[2]^ The immunoblot method is a commonly used semiquantitative approach to measure allergen levels. Globally, the prevalence of hypersensitivity conditions is increasing, presenting a significant health challenge that affects a substantial portion of the population. This rise adversely impacts the well-being of those affected. Hay fever, for instance, a widespread hypersensitivity condition affecting the nasal lining, is estimated to impact between 10% and 40% of people worldwide.^[3–5]^

Over the past 2 decades, China has experienced a marked increase in the prevalence of allergic diseases, a trend closely linked to rapid economic development, urbanization, lifestyle changes, and alterations in dietary habits. Allergic diseases have emerged as one of the prevalent health concerns of the 21st century, affecting an estimated 25% of the global population.^[6]^ These conditions not only deteriorate the quality of life of affected children but can also pose life-threatening risks, leading to a significant economic burden on society. Diagnosing allergic diseases involves a various of methods, each tailored to specific applications and variations in diseases.^[7]^ For type I immediate-type allergic conditions, such as allergic rhinitis, allergic asthma, food allergies, and certain drug allergies, common clinical diagnostic tools include in vivo tests, including prick tests and intradermal tests, along with in vitro tests for the detection of sIgE.^[8]^ Secondary allergen testing is also available as required.

This study aims to examine the levels of sIgE to common pollen and food allergens in patients suffering from localized allergic diseases in the Dongying region of China. Additionally, it will investigate the correlation between these allergen levels and the allergic diseases. The primary objectives of this study are to deepen the understanding of allergic diseases, explore the causes and mechanisms of allergies, strengthen the management of allergic diseases, and provide essential data to support clinical diagnosis and prevention strategies for allergic diseases.

## 2. Materials and methods

### 2.1. Objects

**Figure 1.**
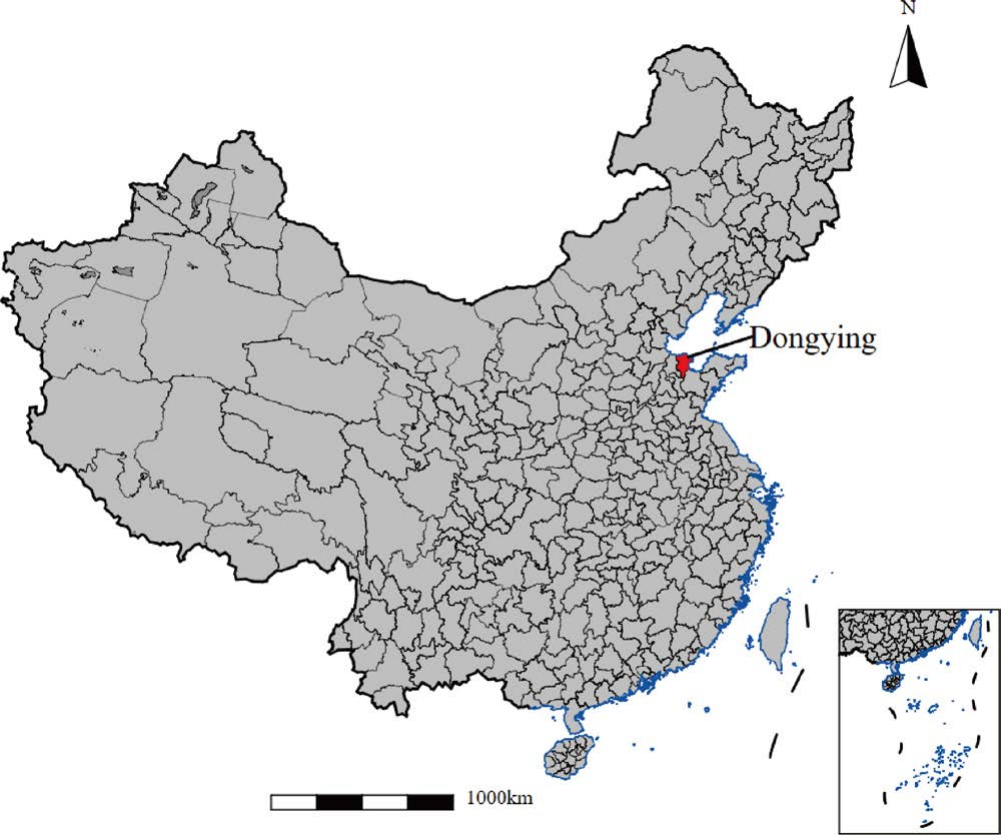
The geographical location of Dongying in China.

This research enrolled individuals presenting with allergies symptoms who sought medical attention at the Allergy Division of Shengli Oilfield Central Hospital between January 2022 and January 2023. The participants included those exhibiting symptoms such as sneezing, nasal itchiness, runny nose, and nasal congestion, indicative of allergic rhinitis, as well as those with signs of asthma such as persistent cough, chest tightness, and b shortness of breath. Additionally, individuals showing skin irritations and eruptions suggestive of eczema were systematically registered at the Allergy Ambulatory Service of Shengli Oilfield Central Hospital. Their sensitivities to specific allergens were determined through sIgE testing. All participants were residents of Dongying, Shandong for at least 1 year (as shown in Fig. 1), and underwent allergen sensitivity testing during their visit to the Allergy Division of Shengli Oilfield Central Hospital. Excluded from the study were non-Dongying natives, individuals diagnosed with nonallergic nasal conditions or growths, and those with medication-induced allergic reactions. This research was conducted in accordance with the Helsinki Declaration and secured approval from the Ethical Review Board of Shengli Oilfield Central Hospital (Q/ZXYY-R-KJK-K/002).

### 2.2. Serum sIgE test

The levels of sIgE in the blood samples from 230 patients were analyzed using the Fobock platform, developed by Jiangsu Haoeubo Company. This platform employs immunoblotting technology for the semiquantitative detection of circulating allergen-sIgE in human serum. To assess sIgE antibody levels, a comprehensive panel of the most common local inhalants and food allergens was used. The allergens common Soybean, wheat flour, house dust mite, pistachio, sycamore, common ragweed, almond, pine, cypress, willow, peach, cottonwood, hazelnut, milk, birch, dust mite, cashew, mugwort, mango, peanut, apple, strawberry, pineapple, humulus, and egg. A concentration of sIgE antibodies equal to or >0.35 IU/mL was considered indicative of a positive sensitization.

### 2.3. Statistical analysis

Data analysis was conducted using the SPSS 25.0 software suite. Differences in gender and age were assessed using the chi-square method. A *P*-value <.05 was deemed to indicate statistical relevance. To visualize the interrelation heatmap of 2 allergens, the ggplot2 tool in R was employed. To gauge the interrelation between 2 allergens for all sIgE positive subjects, the Phi coefficient was utilized; a Phi coefficient of ≥0.70 indicated an extremely high correlation, values ranging from 0.40 to 0.69 indicated a high correlation, values from 0.30 to 0.39 indicated a medium correlation, those from 0.16 to 0.29 were seen as low, and values of ≤0.15 were viewed as trivial.

## 3. Results

### 3.1. Characteristics of the study population

This study encompassed a total of 230 patients suffering from allergic in the Dongying region. The average age of the participants was 16 years. Among these patients, 102 were males, representing 44.35% of the group, while 128 were females, making up 55.65% of the study population. Of the 25 allergens tested, there was 1 to which none of the patients showed sensitization (Table 1).

**Table 1:** Clinical characteristics of patients.

	Overall
Age, year, M ± SD	16.71 ± 15.10
Sex, Male, N (%)	102 (44.35%)
*Allergens, Sensitive population (%*)	
Food allergen	
Wheat Flour	44 (19.13%)
Soybean	6 (2.61%)
Almond	5 (2.17%)
Pistachio	6 (2.61%)
Peach	21 (9.13%)
Milk	18 (7.83%)
Egg	76 (33.04%)
Apple	19 (8.26%)
Strawberry	16 (6.96%)
Pineapple	15 (6.52%)
Mango	4 (1.74%)
Peanut	7 (3.04%)
hazelnut	12 (5.22%)
Pollen	
Cottonwood	22 (9.57%)
Cypress tree	0 (0.0%)
Willow tree	9 (3.91%)
Birch tree	23 (10.0%)
Cashew	8 (3.48%)
Artemisia argyi	46 (20.0%)
Ordinary ragweed	34 (14.78%)
Pine tree	26 (11.3%)
Humulus scandens	37 (16.09%)
Paulownia	27 (11.74%)
Mite	
House dust mite	50 (21.74%)
Dust mite	69 (30.0%)

### 3.2. Prevalence of allergen sensitization among patients

**Figure 2.**
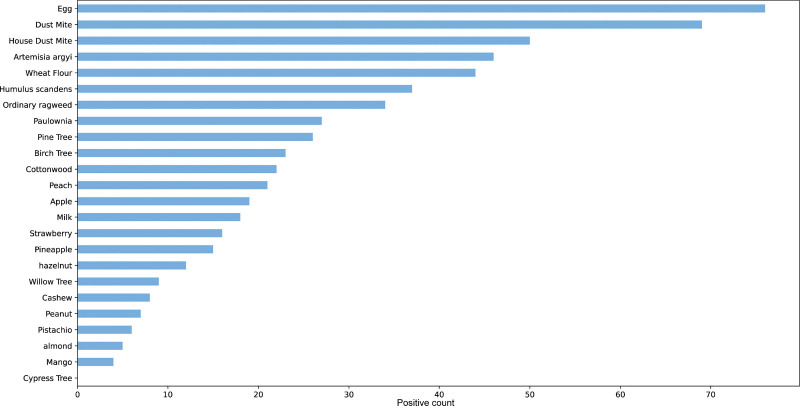
Sort the number of people who are not sensitive to allergens.

We calculated and ranked the sensitization of patients to different allergens. The allergen with the highest number of sensitized individuals was egg, followed closely by dust mite. Conversely, the allergen with the lowest sensitization rate was the cypress tree, with mango ranking just above it. A detailed ranking of the allergens based on the number of sensitized individuals is presented in Figure 2.

### 3.3. Patients with allergen sensitization in different gender groups

**Figure 3.**
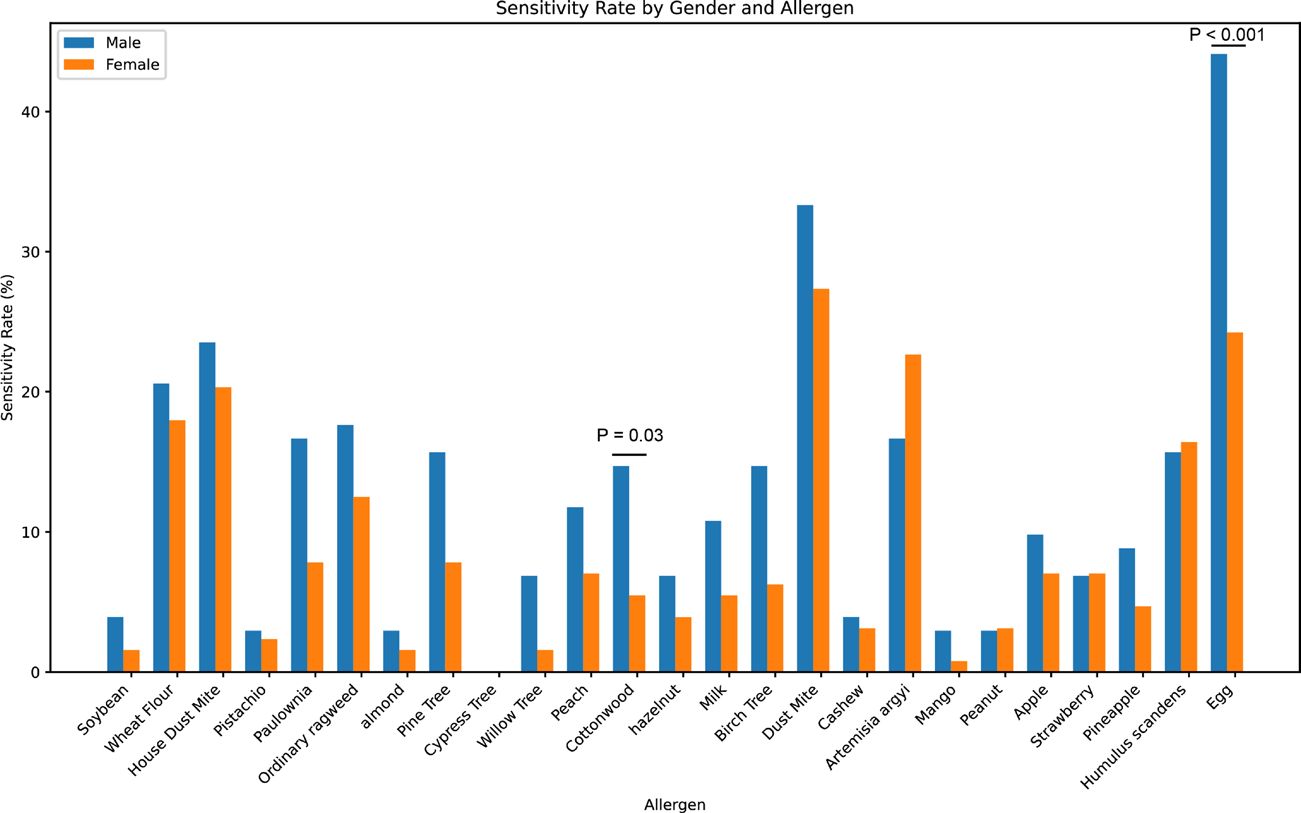
Patients with allergen sensitization in different gender groups.

As illustrated in Figure 3, the study divided patients into male and female groups to explore gender-based differences in allergen sensitivities. Analysis revealed that distinct variations in between genders. Specifically, male patients exhibited higher sensitivities to cottonwood (*P* < .05) and egg (*P* < .001) compared to their female’s counterparts.

### 3.4. Patients with single/multiple positive allergens

**Figure 4.**
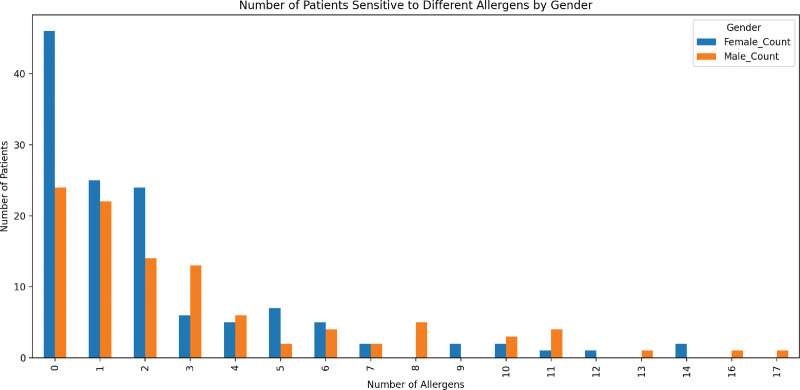
Number of patients sensitive to different allergens by gender.

Patients were categorized according to their sensitivity to single or multiple allergens. Analysis showed that 24 males and 46 females were not sensitive to any of the 25 tested allergens. In contrast, 22 males and 25 females exhibited sensitive to only 1 allergen. Remarkably, 1 male patient showed sensitivity to all 17 allergens, and 2 females patients were sensitive to all 14 allergens. These results are summarized in Table 2 and visualized in Figure 4.

**Table 2:** Percentage of patients sensitized to 1 or more allergens.

Total allergens sensitive	Female count	Male count	Female sensitivity rate	Male sensitivity rate
0	46	24	35.94	23.53
1	25	22	19.53	21.57
2	24	14	18.75	13.73
3	6	13	4.69	12.75
4	5	6	3.91	5.88
5	7	2	5.47	1.96
6	5	4	3.91	3.92
7	2	2	1.56	1.96
8	0	5	0	4.9
9	2	0	1.56	0
10	2	3	1.56	2.94
11	1	4	0.78	3.92
12	1	0	0.78	0
13	0	1	0	0.98
14	2	0	1.56	0
16	0	1	0	0.98
17	0	1	0	0.98

### 3.5. Patients with allergen-sensitization in different age groups

**Figure 5.**
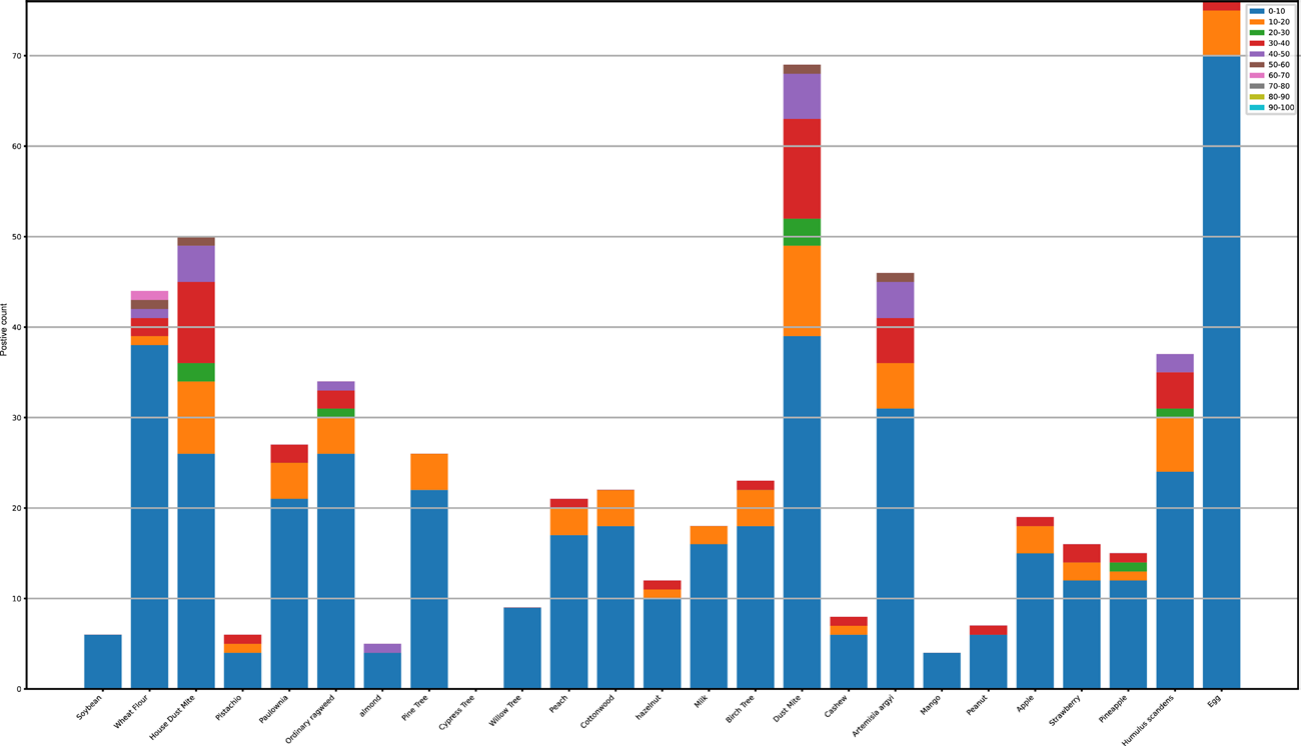
Patients with allergen-sensitization in different age groups.

Patients were also grouped by age, with each group spanning a decade. Analysis highlighted differences in allergen sensitivities among various age groups. Notably, the group aged 0 to 10 years demonstrated higher sensitivity to a range of allergens (Fig. 5).

### 3.6. Correlation between allergens

**Figure 6.**
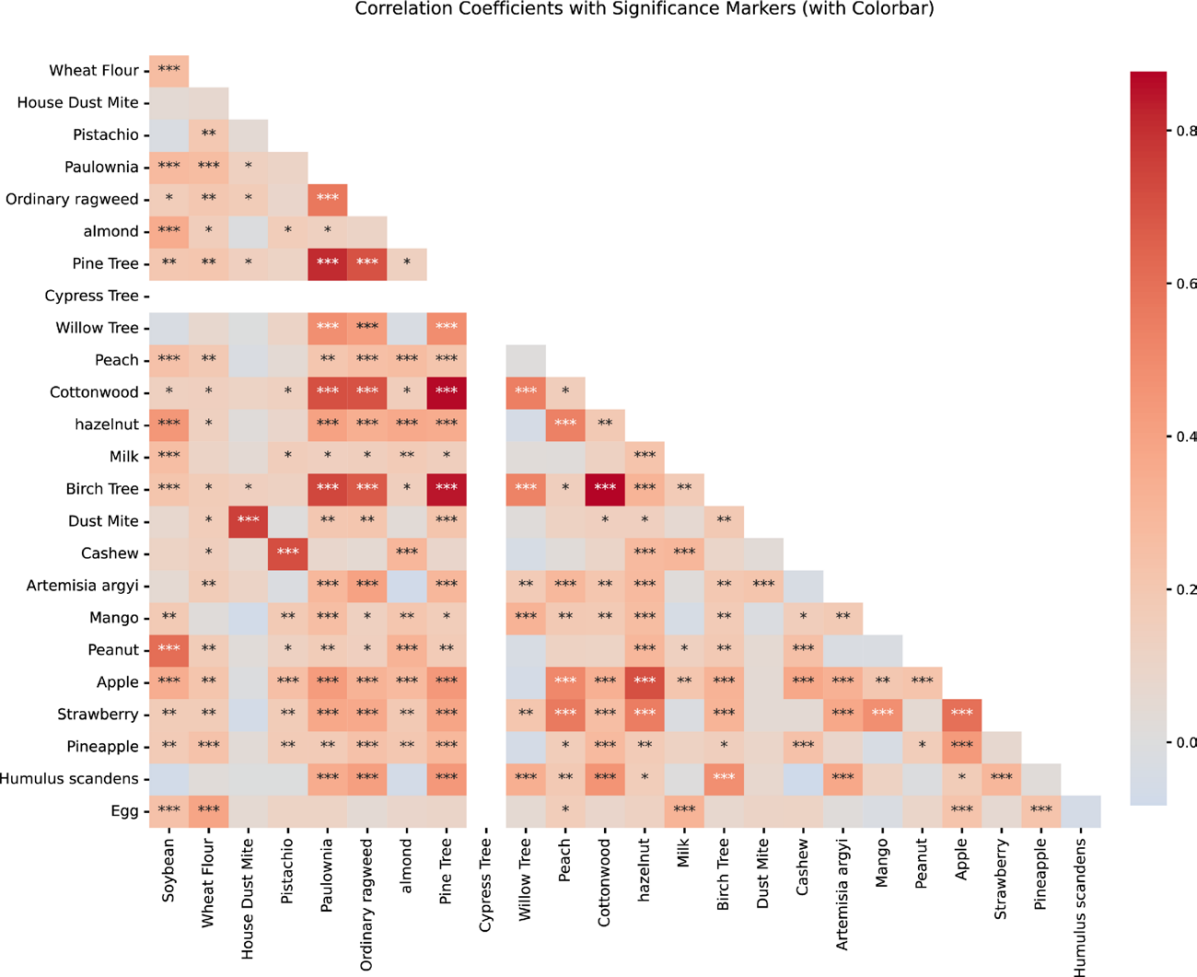
Heatmap of correlation between allergens.

Using the ggplot package in R, we conducted an analysis to assess the correlation between various allergens. Figure 6 revealed significant correlation between different allergens. The top 10 allergen pairs with the highest correlation coefficients were identified as follows: Birch Tree and Cottonwood (0.88, *P* < .001), Cottonwood and Pine Tree (0.86, *P* < .001), Birch Tree and Pine Tree (0.84, *P* < .001), Pine Tree and Paulownia (0.81, *P* < .001), Dust Mite and House Dust Mite (0.76, *P* < .001), Birch Tree and Paulownia (0.73, *P* < .001), Cashew and Pistachio (0.71, *P* < .001), Apple and Hazelnut (0.71, *P* < .001), Cottonwood and Paulownia (0.71, *P* < .001), and Pine Tree and Ordinary Ragweed (0.70, *P* < .001). The detailed data is presented in Table 3.

**Table 3:** Top 10 correlations between allergens.

Allergen1	Allergen2	Correlation	*P*-value
Birch tree	Cottonwood	0.88	<.001
Cottonwood	Pine tree	0.86	<.001
Birch tree	Pine tree	0.84	<.001
Pine tree	Paulownia	0.81	<.001
Dust mite	House dust mite	0.76	<.001
Birch tree	Paulownia	0.73	<.001
Cashew	Pistachio	0.71	<.001
Apple	Hazelnut	0.71	<.001
Cottonwood	Paulownia	0.71	<.001
Pine tree	Ordinary ragweed	0.70	<.001

## 4. Discussion

The World Health Organization (WHO) emphasizes the importance of a comprehensive “four-in-one” optimal treatment plan for patients with allergic diseases. This strategy encompasses the following key components:

China has recently been recognized as a “major country for allergies.” Despite this, the common approach in public hospitals for treating allergic diseases is largely focused on symptomatic medications. While these treatments can offer rapid relief of symptoms, they do not address the chronic nature of allergic diseases.^[9]^ Consequently, patients often experience relapses, as these treatments fail to provide a long-term cure. The World Health Organization (WHO) emphasizes the importance of a comprehensive “four-in-one” optimal treatment plan for patients with allergic diseases.^[10]^ This strategy encompasses the following key components: accurate diagnosis and avoidance of allergens (testing); standardized specific immunotherapy (cause-based treatment); effective patient education (management); and appropriate symptomatic medications (comprehensive medication). Standardized specific immunotherapy stands out as the only treatment that addresses the underlying cause of allergic diseases and offers a long-term management strategy.^[11]^ By conducting localized epidemiological survey of allergens, such as in the Dongying region, healthcare providers can develop more comprehensive and effective treatment and management plans tailored to the specific needs of allergy patients.^[12]^

Dongying, situated in the central part of Shandong Province and near the Yellow River Delta, experiences a temperate monsoon climate with distinct seasonal changes.^[13]^ The winters here are notably cold, while summers bring intense heat, and the spring and autumn seasons present moderate conditions. Given these geographical and climatic attributes, it is possible to infer certain epidemiological trends among the allergic population in the region.^[14]^ The prominent feature of a temperate monsoon climate is a pronounced spring season, which is typically associated with peak pollen dispersion. This suggests that pollen allergies could be a major health concern for for residents in Dongying.^[15]^ During this season, trees, grasses, and weeds release large amounts of pollen, potentially leading to an increase in cases of seasonal allergic rhinitis, asthma, and other allergic reactions.^[16]^ Furthermore, Dongying relatively high humidity levels create an ideal environment for the proliferation of dust mites.^[17]^ These microscopic organisms are a primary source of indoor allergens and thrive in humid conditions. Their presence is a significant factor in triggering allergic reactions such as allergic rhinitis, asthma, and skin-related symptoms in individuals who are sensitive to these allergens.^[18]^

The fluctuating humidity and temperature levels in Dongying might also encourage mold growth, particularly in damp environments. Mold spores, when airborne, can become allergens that trigger respiratory and skin reactions in individuals allergic to them.^[19]^ While food allergies may not be directly linked to geographical location or climate, regional dietary habits can influence the prevalence of specific food allergies.^[20]^ For instance, a higher consumption of certain foods in a region could lead to increased incidences of allergic reactions to those food.

Additionally, households in Dongying with pets might experience allergic reactions to pet dander, urine, and saliva.^[21]^ It is also important to consider the impact of industrial zones in Dongying. Industrial pollution could exacerbate certain allergic symptoms, especially respiratory ailments.^[22]^ The interplay of geography, climate, and lifestyle in Dongying suggests that allergic population in the area may be influenced by a myriad of factors. These factors natural pollen dispersion from local vegetation and human-made pollutants from industrial activities.^[23–25]^

Dongying unique profile, characterized by its temperate monsoon climate, proximity to the Yellow River Delta, and a blend of urban and industrial areas, creates a complex tapestry of allergenic challenges.^[26,27]^ For individuals susceptible to allergies, understanding these challenges is crucial.^[28,29]^ However, to gain a more accurate insight into the epidemiological characteristics of allergies in Dongying, in-depth research and surveys would be indispensable.^[30]^ The findings from such studies could then inform public health strategies, ensuring that residents and visitors are well-equipped to manage and mitigate allergic reactions effectively.^[31]^

This study involving 230 allergy patients, showed an almost equal distribution between males and female participants. Among the allergen tested, egg triggered the highest sensitization, followed by dust mite, while cypress tree and mango were the least sensitizing allergens. Notably, gender differences in allergen sensitivity were observed; male patients had higher sensitivities to cottonwood and egg compared to females, suggesting distinct gender-based variations in response to specific allergens. Additionally, the study stratified patients into different age groups, patients into different age, with intervals of 10 years, to analyze the differences in allergen sensitivities across age groups groups, with ten-year intervals, to examine allergen sensitivities across various age brackets. A key finding was the heightened allergen sensitivity in the 0 to 10 years age group. This indicates a higher propensity for developing allergies among children, underscoring the need to prioritize them in prevention strategies. The analysis of the correlation between different allergens revealed significant associations, suggesting that an individual’s sensitivity to allergens may be more closely tied to their overall allergic predisposition. Individuals who are inherently prone to allergies are likely to exhibit sensitivities across a broader spectrum of allergens. This insight into the complex nature of allergen sensitivities highlights the importance of personalized approaches in the diagnosis, treatment, and management of allergic conditions.

In summary, this investigation offers new insights into the prevalence of allergen reactions in Dongying, Shandong, highlighting egg as the most common allergen. T A significant correlation was observed between between Birch Tree and Cottonwood. Unlike previous studies in the Chinese context, which often relied on self-reported data, this research offers a factual analysis of sensitization frequencies to a variety of allergens among individuals in Dongying, potentially suffering from allergic conditions. The findings could be relevant to other northern Chinese provinces with similar environmental and patient behaviors patterns. Nonetheless, our study focused on a limited range of allergens, excluding several common ones. Additionally, being a single-institution retrospective analysis, the findings are based on a specific patient set and location. To enhance the reliability and applicability of these results, further comprehensive, prospective studies across multiple institutions are needed. These studies should aim to understand the prevalence and nature of allergic reactions in Dongying and other regions in China, contributing to better identification and treatment strategies for allergies. A notable limitation of this study is the lack of recorded symptoms in the allergic patients. This gap hinders a thorough understanding of the relationship between specific allergens and the symptoms they induce, as well as the potential variations in symptoms caused by different allergens. Future research efforts will be directed towards a detailed investigation of the symptoms associated with various allergens, which is expected to have significant clinical implications. This approach will enable a more nuanced understanding of allergic reactions, facilitating more effective management and treatment of allergic conditions.

## 5. Conclusion

It has also contributed to improving the management of allergic conditions and laid a foundation for the clinical diagnosis and prevention of these diseases. This work represents a significant step forward in the field of allergy research and patient care.

This research provides valuable insights into the prevalence of sensitization to various allergens in Dongying, Shandong, identifying egg as the most prevalent sensitizing allergen. A notable correlation was observed between Birch Tree and Cottonwood allergens. This study has enriched our understanding of allergic diseases, explored the causes and mechanisms of allergies. It has also contributed to improving the management of allergic conditions and laid a foundation for the clinical diagnosis and prevention of these diseases. This work represents a significant step forward in the field of allergy research and patient care.

## Author contributions

**Conceptualization:** Yingying Zhang, Lianjun Gao.

**Data curation:** Yingying Zhang, Mei Shang, Lianjun Gao.

**Formal analysis:** Mei Shang, Ying Tian, Xuwei Liu.

**Funding acquisition:** Yingying Zhang, Lianjun Gao.

**Investigation:** Xuhui Sun.

**Methodology:** Yingying Zhang, Mei Shang, Xuhui Sun, Lianjun Gao.

**Project administration:** Yingying Zhang, Lianjun Gao.

**Resources:** Yingying Zhang.

**Software:** Yingying Zhang, Lianjun Gao.

**Supervision:** Yingying Zhang, Lianjun Gao.

**Validation:** Yingying Zhang.

**Visualization:** Yingying Zhang, Lianjun Gao.

**Writing – original draft:** Yingying Zhang.

**Writing – review & editing:** Yingying Zhang, Lianjun Gao.

## References

[1] Kausar MA Bhardwaj T Anwar S. In silico comparative exploration of allergens of periplaneta americana, blattella germanica and phoenix dactylifera for the diagnosis of patients suffering from IgE-mediated allergic respiratory diseases. Molecules. 2022;27:8740. 36557872 10.3390/molecules27248740 PMC9785491

[2] Breiteneder H Peng Y-Q Agache I. Biomarkers for diagnosis and prediction of therapy responses in allergic diseases and asthma. Allergy. 2020;75:3039–68. 32893900 10.1111/all.14582 PMC7756301

[3] Howarth PH Persson CGA Meltzer EO. Objective monitoring of nasal airway inflammation in rhinitis. J Allergy Clin Immunol. 2005;115:S414–441. 15746881 10.1016/j.jaci.2004.12.1134

[4] Arruda LK Vailes LD Ferriani VP. Cockroach allergens and asthma. J Allergy Clin Immunol. 2001;107:419–28. 11240940 10.1067/mai.2001.112854

[5] Zhang Y Zhang L. Increasing prevalence of allergic rhinitis in China. Allergy Asthma Immunol Res. 2019;11:156–69. 30661309 10.4168/aair.2019.11.2.156 PMC6340797

[6] Fernández-Caldas E Cases B El-Qutob D. Mammalian raw materials used to produce allergen extracts. Ann Allergy Asthma Immunol. 2017;119:1–8. 28668236 10.1016/j.anai.2016.08.024

[7] Chapman MD Briza P. Molecular approaches to allergen standardization. Curr Allergy Asthma Rep. 2012;12:478–84. 22740009 10.1007/s11882-012-0282-3

[8] Mullol J Valero A Alobid I. Allergic Rhinitis and its Impact on Asthma update (ARIA 2008) the perspective from Spain. J Investig Allergol Clin Immunol. 2008;18:327–34. 18973095

[9] Kosti RI Triga M Tsabouri S. Food allergen selective thermal processing regimens may change oral tolerance in infancy. Allergol Immunopathol (Madr). 2013;41:407–17. 23253679 10.1016/j.aller.2012.08.011

[10] Tomazic-Jezic VJ Lucas AD. Protein and allergen assays for natural rubber latex products. J Allergy Clin Immunol. 2002;110:S40–46. 12170242 10.1067/mai.2002.125335

[11] Radauer C Nandy A Ferreira F. Update of the WHO/IUIS allergen nomenclature database based on analysis of allergen sequences. Allergy. 2014;69:413–9. 24738154 10.1111/all.12348

[12] Calzada D Iraola V Carnés J. Heterogeneity of allergen content in male dog urine and dander. J Investig Allergol Clin Immunol. 2020;30:213–4. 10.18176/jiaci.0487 31983678

[13] Sicherer SH Sampson HA. Food allergy: epidemiology, pathogenesis, diagnosis, and treatment. J Allergy Clin Immunol. 2014;133:291–307; quiz 308. 24388012 10.1016/j.jaci.2013.11.020

[14] Sicherer SH Sampson HA. Food allergy: a review and update on epidemiology, pathogenesis, diagnosis, prevention, and management. J Allergy Clin Immunol. 2018;141:41–58. 29157945 10.1016/j.jaci.2017.11.003

[15] Warren CM Jiang J Gupta RS. Epidemiology and burden of food allergy. Curr Allergy Asthma Rep. 2020;20:6. 32067114 10.1007/s11882-020-0898-7 PMC7883751

[16] Nelson HS. Allergen immunotherapy: where is it now? J Allergy Clin Immunol. 2007;119:769–79. 17337297 10.1016/j.jaci.2007.01.036

[17] Larsen JM Bang-Berthelsen CH Qvortrup K. Production of allergen-specific immunotherapeutic agents for the treatment of food allergy. Crit Rev Biotechnol. 2020;40:881–94. 32515236 10.1080/07388551.2020.1772194

[18] Valenta R Kraft D. From allergen structure to new forms of allergen-specific immunotherapy. Curr Opin Immunol. 2002;14:718–27. 12413521 10.1016/s0952-7915(02)00402-8

[19] Chan SK Pomés A Hilger C. Keeping allergen names clear and defined. Front Immunol. 2019;10:2600. 31798576 10.3389/fimmu.2019.02600 PMC6878850

[20] Schubert M Spiegel H Schillberg S. Aspergillus-specific antibodies - Targets and applications. Biotechnol Adv. 2018;36:1167–84. 29608951 10.1016/j.biotechadv.2018.03.016

[21] Becker W-M Vogel L Vieths S. Standardization of allergen extracts for immunotherapy: where do we stand? Curr Opin Allergy Clin Immunol. 2006;6:470–5. 17088654 10.1097/01.all.0000246622.34247.21

[22] Carnés J Iraola V Cho SH. Mite allergen extracts and clinical practice. Ann Allergy Asthma Immunol. 2017;118:249–56. 28284531 10.1016/j.anai.2016.08.018

[23] Greiner AN Hellings PW Rotiroti G. Allergic rhinitis. Lancet. 2011;378:2112–22. 21783242 10.1016/S0140-6736(11)60130-X

[24] Zhang Y Lan F Zhang L. Advances and highlights in allergic rhinitis. Allergy. 2021;76:3383–9. 34379805 10.1111/all.15044

[25] Dykewicz MS Wallace DV Baroody F. Treatment of seasonal allergic rhinitis: an evidence-based focused 2017 guideline update. Ann Allergy Asthma Immunol. 2017;119:489–511.e41. 29103802 10.1016/j.anai.2017.08.012

[26] Radauer C. Navigating through the jungle of allergens: features and applications of allergen databases. Int Arch Allergy Immunol. 2017;173:1–11. 28456806 10.1159/000471806

[27] Lou H Huang Y Ouyang Y. Artemisia annua-sublingual immunotherapy for seasonal allergic rhinitis: a randomized controlled trial. Allergy. 2020;75:2026–36. 32030780 10.1111/all.14218

[28] Bellanti JA Settipane RA DuBuske L. Essentials of allergen immunotherapy: a primer for the practitioner. Allergy Asthma Proc. 2022;43:245–7. 35818156 10.2500/aap.2022.43.220039 PMC9274934

[29] Gerblich AA Schwartz HJ Chester EH. Seasonal variation of airway function in allergic rhinitis. J Allergy Clin Immunol. 1986;77:676–81. 3700890 10.1016/0091-6749(86)90408-2

[30] David NA Penumarti A Burks AW. Food allergen extracts to diagnose food-induced allergic diseases: how they are made. Ann Allergy Asthma Immunol. 2017;119:101–7. 28801015 10.1016/j.anai.2016.11.008

[31] Linacero R Sanchiz A Ballesteros I. Application of real-time PCR for tree nut allergen detection in processed foods. Crit Rev Food Sci Nutr. 2020;60:1077–93. 30638046 10.1080/10408398.2018.1557103

